# Oleuropein has hypophagic effects in broiler chicks

**DOI:** 10.3389/fphys.2024.1409211

**Published:** 2024-06-12

**Authors:** Usman Sulaiman, Reagan Vaughan, Paul Siegel, Dongmin Liu, Elizabeth Ruth Gilbert, Mark Andrew Cline

**Affiliations:** ^1^ School of Animal Sciences, Virginia Polytechnic Institute and State University, Blacksburg, VA, United States; ^2^ Department of Human Nutrition, Foods and Exercise, Virginia Polytechnic Institute and State University, Blacksburg, VA, United States; ^3^ School of Neuroscience, Virginia Polytechnic Institute and State University, Blacksburg, VA, United States

**Keywords:** oleuropein, broiler, feed intake, dietary supplement, blood glucose

## Abstract

Oleuropein, a phenolic compound derived from olives, has known glucoregulatory effects in mammalian models but effects in birds are unknown. We investigated effects of dietary supplementation and exogenous administration of oleuropein on broiler chick feed intake and glucose homeostasis during the first 7 days post-hatch. One hundred and forty-eight day-of-hatch broiler chicks were randomly allocated to one of four dietary treatments with varying oleuropein concentrations (0, 250, 500, or 1,000 mg/kg). Body weight and breast muscle and liver weights were recorded on day 7. In the next experiment, chicks received intraperitoneal (IP) injections of oleuropein at doses of 0 (vehicle), 50, 100, or 200 mg/kg on day 4 post-hatch, with feed intake and blood glucose levels measured thereafter. Lastly, chicks fed a control diet were fasted and administered intracerebroventricular (ICV) injections of oleuropein at doses of 0, 50, 100, or 200 μg, after which feed intake was recorded. Results indicated that IP and ICV injections led to decreased feed intake, primarily at 60 min post-injection, with effects diminishing by 90 min in the IP study. Blood glucose levels decreased 1-h post-IP injection at higher oleuropein doses. These findings suggest that oleuropein acts as a mild appetite suppressant and influences energy metabolism in broiler chickens.

## 1 Introduction

Polyphenolic phytochemicals, naturally occurring metabolites found in plants, have garnered increasing attention due to their potential to elicit a wide range of beneficial physiological effects in both humans and animals, including anti-inflammatory, anti-oxidative, pro-gut health, and metabolic responses (Starčević et al., 2015; Shimao et al., 2019). As a result, plant extracts and plant-derived polyphenolic compounds have emerged as novel feed ingredients with the capacity to modulate growth and enhance overall health across several species. Given the phasing out of antibiotics at sub-therapeutic levels in poultry diets, identifying alternatives that exert health- and growth-promoting properties is important to the poultry industry and consumers of poultry products.

Oleuropein, a key component of extra virgin olive oil (EVO) with higher phytochemical content than pure olive oil (POO), has demonstrated health benefits in both human subjects and animals (Alkhatib et al., 2018). It was suggested that the beneficial metabolic effects of EVO, distinguished by its nearly identical fatty acid composition to POO, but substantially higher phenolic content, are in part due to its phenolic components (Bogani et al., 2007). Among these components, oleuropein, a non-toxic glycosylated seco-iridoid phenol extracted from olives, holds particular promise in modulating physiological processes or functions. Oleuropein’s abundance in olives and olive leaves, coupled with its high bioavailability, rapid absorption, and maximal plasma concentrations within 2 h after oral administration in humans, underscore its potential as a novel food ingredient (Omar, 2010).

Oleuropein’s physiological impact has been investigated in several mammalian species including humans and mice, demonstrating a spectrum of benefits including antioxidant, antimicrobial, anticancer, hypoglycemic, hypolipidemic, anti-inflammatory, antiatherogenic, and antiviral effects (Omar, 2010; Haidari et al., 2021). In addition, oleuropein has shown the potential to prevent hepatic steatosis in rodent models (Park et al., 2011), a condition of particular concern in the poultry layer industry, where fatty liver disease affects laying hens (Lin et al., 2021).

In light of the potential benefits of oleuropein, this study seeks to investigate its effects on broiler chickens, specifically focusing on breast muscle and liver weight, as well as variations in feed intake and blood glucose levels. Our research aims to contribute insights into the physiological impacts of oleuropein in broilers, with potential implications for poultry production and animal welfare.

## 2 Methods

### 2.1 Animals

All animal protocols were approved by the Institutional Animal Care and Use Committee (IACUC) at Virginia Tech. One hundred and forty-eight Hubbard x Cobb-500 broiler chicks were obtained from a nearby commercial hatchery on the day of the hatch. Upon arrival at our facility, they were maintained at 30°C ± 2°C and 50% ± 5% relative humidity with free access to feed and water.

### 2.2 Experiment 1: dietary oleuropein supplementation

Forty-four chicks were assigned to one of four diets that contained oleuropein at 0, 250, 500, or 1,000 mg/kg. Dietary inclusion levels were based on a previous study involving plant compound supplementation, with a higher “high” dose to identify the effective dose range (Xiao et al., 2021). Oleuropein was purchased from Xi’An Yile Bio-Tech Company, China, and was tested for purity using high-performance liquid chromatography (HPLC, purity ≥90%).

Daily body weight data were collected for all broiler chicks. On day 7 post-hatch, breast muscle and liver weights were recorded. To assess organ weight relative to body weight, we computed the ratio of organ weight to the corresponding body weight. Data are reported for male chicks.

### 2.3 Experiment 2: peripheral oleuropein administration

Fifty-two day-of-hatch chicks were fed the control diet and on day 2 post-hatch, they were housed in individual cages that provided visual and auditory contact with other chicks and permitted measurement of individual feed intake. On day 4 post-hatch, 48 chicks (*n* = 12 per treatment) were randomly assigned to receive an intraperitoneal (IP) injection of one of four doses of oleuropein diluted in saline (0, 50, 100, or 200 mg/kg of body weight) and delivered in a volume of 150 μL with insulin syringes (BD Biosciences). The injection technique was described by Rice et al. (2014). Doses were based on previous studies with plant compounds, with a higher dose used to identify the effective dose range (Xiao et al., 2021). Feed was withheld before injection for 3 h. After injection, chicks were returned to individual cages, and feed intake was measured (to the nearest 0.1 g) in 30-min intervals for 90 min. Chicks were then euthanized by cervical dislocation, and sex was determined via gonadal inspection.

### 2.4 Experiment 3: central oleuropein administration

Housing and diets were the same as in Experiment 2. On day 4 post-hatch, 52 chicks were randomly assigned to receive an intracerebroventricular (ICV; in the left lateral ventricle of the brain) injection of one of four doses of oleuropein diluted in artificial cerebrospinal fluid (0, 50, 100, and 200 μg). Forty-eight chicks (*n* = 12 per treatment) were injected using a method adapted from Davis et al. (1979) that does not appear to induce physiological stress and is used routinely by our group. The heads of the chicks were briefly inserted into a restraining device that left the cranium exposed and allowed for freehand injection with a Hamilton syringe. Injection coordinates were 3 mm anterior to the coronal suture, 1 mm lateral from the sagittal suture, and 2 mm deep targeting the left lateral ventricle. Anatomical landmarks were determined visually and by palpation. Injection depth was controlled by placing a plastic tubing sheath over the needle. The needle remained at injection depth in the un-anesthetized chick for 5 s post-injection to reduce backflow. The total injection volume was 5 μL and contained 0.06% Evans Blue dye to facilitate injection site localization after the experiment. After injection, chicks were returned to their individual cages, and feed intake (to the nearest 0.1 g) was recorded in 30-min intervals for 90 min. After data collection, the chicks were decapitated and their heads sectioned along the frontal plane to determine the site of injection. Any chick without dye present in the lateral ventricle system was eliminated from the analysis. Sex was determined via gonadal inspection.

### 2.5 Experiment 4: blood glucose

On day 4 post-hatch, 40 chicks fed a standard starter diet were administered oleuropein intraperitoneally using four doses (0, 50, 100, and 200 mg/kg of body weight). Blood glucose levels were measured at 1-h post-injection using hand-held glucometers (Sumners et al., 2014).

### 2.6 Data analysis

Body weight gain, organ weight, blood glucose, and feed intake data were analyzed by ANOVA using the Fit Model platform of JMP Pro 16 (SAS Institute Inc., Cary, NC, 1989–2023). The model included the main effect of treatment within time and Tukey’s test was used for *post hoc* pairwise comparisons and results with a *p*-value ≤0.05 were considered significant. Initial ANOVAs for Experiments 2 and 3 included sex in the model; however, sex was not significant and removed as a variable from subsequent models.

## 3 Results

### 3.1 Growth performance and organ weights

There was no effect on body weight gain during the first-week post-hatch. Average daily gain throughout the experiment did not differ among groups (data not shown) and was not different at day 7 (Table 1). Similarly, breast muscle and liver were similar among treatment groups on day 7.

**TABLE 1:** The effect of dietary oleuropein supplementation on body weight gain and organ weights at day 7 post-hatch in Hubbard × Cobb-500 broilers^a^.

Oleuropein dose	Average daily weight gain to day 7 (g)	Liver weight as % of body weight	Breast muscle weight as % of body weight
0	29.54	4.00	11.60
250	30.38	4.01	11.88
500	28.83	3.78	12.22
1,000	32.80	3.93	12.82
SEM	1.63	0.13	0.13
*p*-value	0.34	0.57	0.22

^a^ Values represent least squares means and pooled standard errors of the means with associated *p*-values for the effect of dietary treatment (*n* = 10/group; males). Treatments are dietary inclusion level of oleuropein (mg/kg).

### 3.2 Feed intake after peripheral and central administration

**FIGURE 1:**
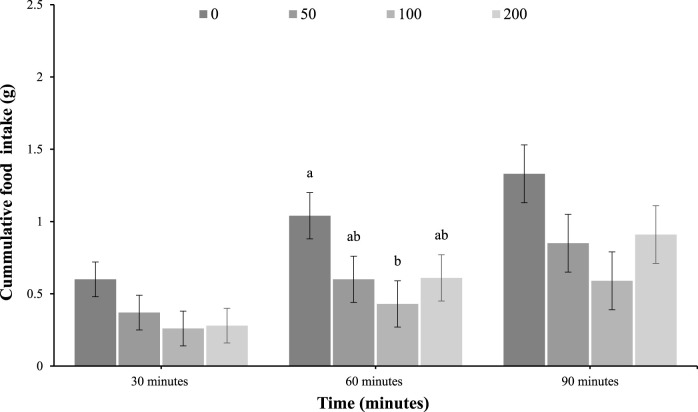
Feed intake of 4-day-old broiler chicks after intraperitoneal (IP) injection with 0 (vehicle), 50, 100, or 200 mg/kg of body weight oleuropein. Chicks were fed a standard starter diet *ad lib* after hatch (*n* = 12 per group; mixed sex). Different letters within each time indicate a difference at *p* < 0.05; Tukey’s test (main effect of treatment at 60 min; *p* = 0.05).

As shown in Figure 1, feed intake was not different among IP-injected groups at 30 min (*p* = 0.22) but was reduced in response to the 100 mg/kg of oleuropein dose at 60 min (main effect of treatment; *p* = 0.05), and was not different at 90 min post-injection (*p* = 0.11).

**FIGURE 2:**
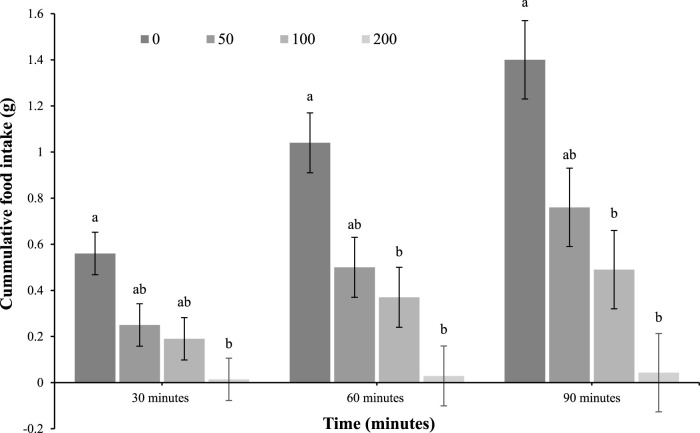
Feed intake of 4-day-old broiler chicks after intracerebroventricular (ICV) injection with 0 (vehicle), 50, 100, or 200 μg oleuropein. Chicks were fed a standard starter diet *ad lib* after hatch (*n* = 12 per group; mixed sex). Different letters within each time indicate a difference at *p* < 0.05; Tukey’s test (main effect of treatment at each time point; *p* < 0.05).

In contrast, there was a reduction in feed intake in response to the highest ICV dose (200 mg/kg) at 30 min post-injection (main effect of treatment; *p* = 0.02), while the 100 and 200 mg/kg doses were effective at 60 (main effect; *p* = 0.002) and 90 (main effect; *p* = 0.0005) minutes (Figure 2).

### 3.3 Blood glucose

**FIGURE 3:**
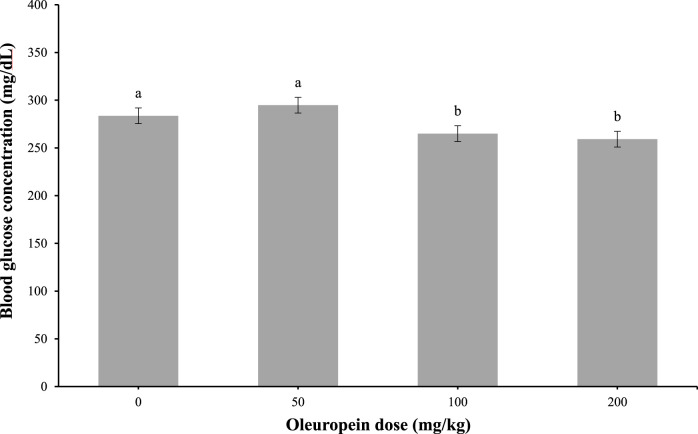
Whole blood glucose in fasted chicks at 1-h post-intraperitoneal injection with 0 (vehicle), 50, 100, or 200 mg/kg of body weight oleuropein. Chicks were fed a standard starter diet *ad lib* (*n* = 10 per group; mixed sex). Main effect of treatment, *p* = 0.0479. Different letters within each sampling measurement indicate a significant difference at *p* < 0.05; Tukey’s test.

At 1-h post-IP injection, 100 and 200 mg/kg of oleuropein doses were efficacious (main effect; *p* = 0.04) at lowering blood glucose concentrations in chicks (Figure 3). The control and the 50 mg/kg of oleuropein dose values were similar.

## 4 Discussion

The objective of this experiment was to determine the effects of oleuropein on broiler chick growth and feed intake during the first week post-hatch. Body weight data were consistent with those of Shimao et al. (2019) who reported that dietary oleuropein supplementation did not impact final body weights at day 22 and that there was no difference in body weight gain and feed efficiency between days 8–22 in Ross 308 broilers. However, it is noteworthy that the liver weight was not affected by dietary oleuropein. This suggests that oleuropein’s impact may not necessarily extend to the liver which is a repository for fat, particularly in older chickens.

Two experiments were designed to directly determine the effects of oleuropein on feed intake. Oleuropein was administered intraperitoneally and intracerebroventricularly because of variability in feed consumption (timing and amount) in an *ad lib* feeding setting. We injected oleuropein peripherally–a route that bypasses the effects of the gastrointestinal tract and can assess the contributions of peripheral factors to central appetite regulation in the animal. The chicks were fasted for 3 h prior to injection to intensify their hunger so that differences were more easily detected using fewer chicks. The hypophagic effects were relatively mild and delayed, with reductions observed in response to the low dose at 60 min post-injection.

Because of the peripheral route coupled with the time taken to observe an effect (>30 min), it is possible that effects on feed intake via the IP route were indirect, with oleuropein affecting some secondary pathway that caused the reduced feed intake. Also, it is possible that oleuropein is bio-transformed after IP injection (e.g., in the liver) and its metabolites exert an appetite regulatory effect. In humans, oleuropein is very bioavailable, however, it is metabolized to tyrosol and hydroxytyrosol in the blood or liver, which are excreted in the urine (Vissers et al., 2002). Thus, to determine whether oleuropein can exert direct effects on appetite, and to avoid confounds from peripheral injection, in the next experiment, oleuropein was intracerebroventricularly (ICV; in the left lateral ventricle of the brain) injected into the chick. Via this route, the ventricle system of the brain then delivers the oleuropein directly to the hypothalamus, the brain region primarily responsible for homeostatic feed intake. We found that when oleuropein was injected directly into the brain, feed intake was acutely suppressed within 30 min post-injection, with almost complete cessation in response to the highest dose. These findings support the notion that oleuropein directly influences appetite regulation in broilers. This is in contrast to the findings of Amini et al. (2019) who reported that dietary-supplemented olive leaf extract (OL) did not significantly affect feed intake across treatment groups. However, they reported that chickens receiving the 10,000 mg/kg OL ate less than those that received the 2,500 mg/kg OL dose. The contrasting results of Amini et al. (2019) can be attributed to differences in compound composition and purity (i.e., extract vs pure compound), dosage levels, administration routes, and the potential variations in compound bioavailability and experimental conditions.

Comparing the two routes of administration, the percentage reduction in feed intake was more pronounced and immediate with ICV than IP injections. The ICV injections led to a nearly complete cessation of feed intake at the highest dose within 30 min, whereas the IP injections induced a milder reduction, primarily observed at 60 min post-injection. This suggests that the central administration of oleuropein has a stronger and faster impact on suppressing appetite compared to peripheral administration. The difference in effect could be due to the primary mode of action of the chemical (e.g., binding sites in central nervous system vs periphery) and whether the peripherally-injected chemical crosses the blood-brain barrier which may not be completely closed at the ages studied, and potential biotransformation of oleuropein in the liver as described above. That both routes induced a reduction in feed intake suggests that oleuropein affects appetite-regulatory pathways in the bird.

While the direct appetite-regulating effects of oleuropein are novel in any species, our glucoregulatory results align with the findings of Jung et al. (2019) who indicated a blood glucose-lowering and insulin sensitivity-improving effect of olive leaf extract in a mouse model. In our experiment, we observed that the two higher doses of oleuropein significantly lowered blood glucose concentrations in broiler chicks at 1-h post-injection. Importantly, this glucose-lowering effect was not a consequence of reduced feed intake, as the chicks were fasted before injection, and feed was withheld after injection. As chickens, like other avians, are inherently hyperglycemic with blood glucose levels that are particularly robust in response to fasting (Rice et al., 2014), the acute effect of oleuropein on blood glucose in broiler chicks is striking.

Although beneficial health effects are attributed to the consumption of olive oil and olive oil extracts, the chemicals that confer these bioactivities are still unclear and the mechanisms underlying effects on appetite and metabolism are also unknown. Thus, this research can further the use of oleuropein as a novel feed ingredient and dietary supplement across a range of species. In particular, this is the first report to our knowledge of a direct appetite-regulatory effect of oleuropein. Collectively, our results suggest that in chickens, oleuropein is a mild appetite suppressant that affects metabolic pathways related to glucose homeostasis, and thus may act to redirect energy during the post-absorptive state from the adipose tissue to the skeletal muscle, which should be addressed in the future studies. Further research is needed to elucidate the underlying mechanisms of the appetite- and glucose-regulatory effects and to optimize oleuropein supplementation strategies for practical application in poultry production.

## Data Availability

The raw data supporting the conclusions of this article will be made available by the authors, without undue reservation.
